# Cutaneous leishmaniasis lowers the quality of life: a neglected truth

**DOI:** 10.3205/dgkh000447

**Published:** 2023-09-21

**Authors:** Nader Aghakhani, Mehdi Azami, Mohammad Ali Mohaghegh

**Affiliations:** 1 Food and Beverages Safety Research Center, Urmia University of Medical Sciences, Urmia, Iran; 2 Skin Diseases and Leishmaniosis Research Center, Isfahan University of Medical Sciences, Isfahan, Iran; 3 Department of Medical Parasitology and Microbiology, Hojjatieh Medical Diagnostic Laboratory, Hojjatieh Hospital, Isfahan, Iran; 4 Basir Laboratory Research and Development Center, Basir Medical Diagnostic Laboratory, Isfahan, Iran; 5 Department of Laboratory Sciences, School of Paramedical Sciences, Torbat Heydariyeh University of Medical Sciences, Torbat Heydariyeh, Iran; 6 Health Sciences Research Center, Torbat Heydariyeh University of Medical Sciences, Torbat Heydariyeh, Iran

## Letter to the editor

**Figure 1:**
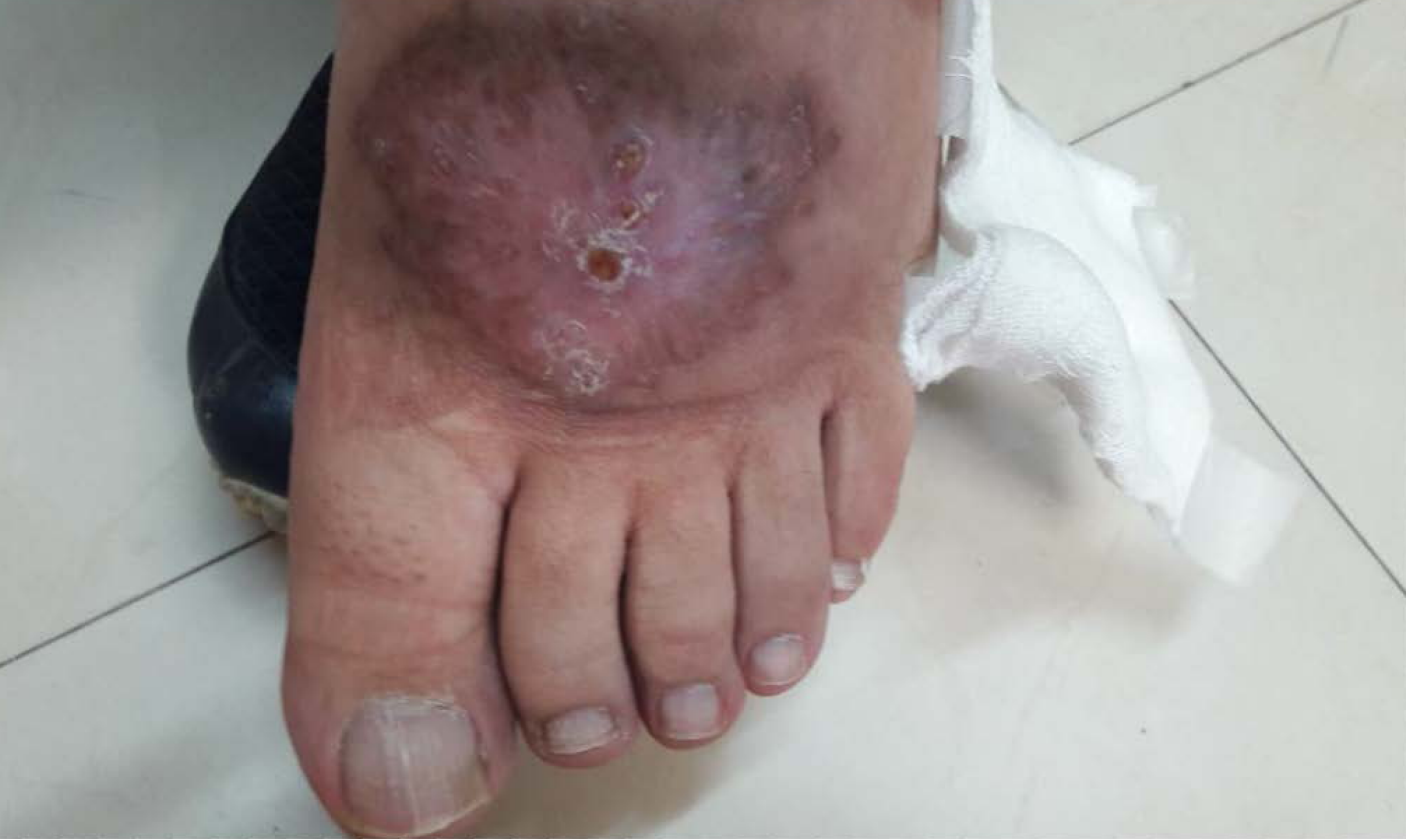
Figue 1: Cutaneous leishmaniasis (Cl), example 1

**Figure 2:**
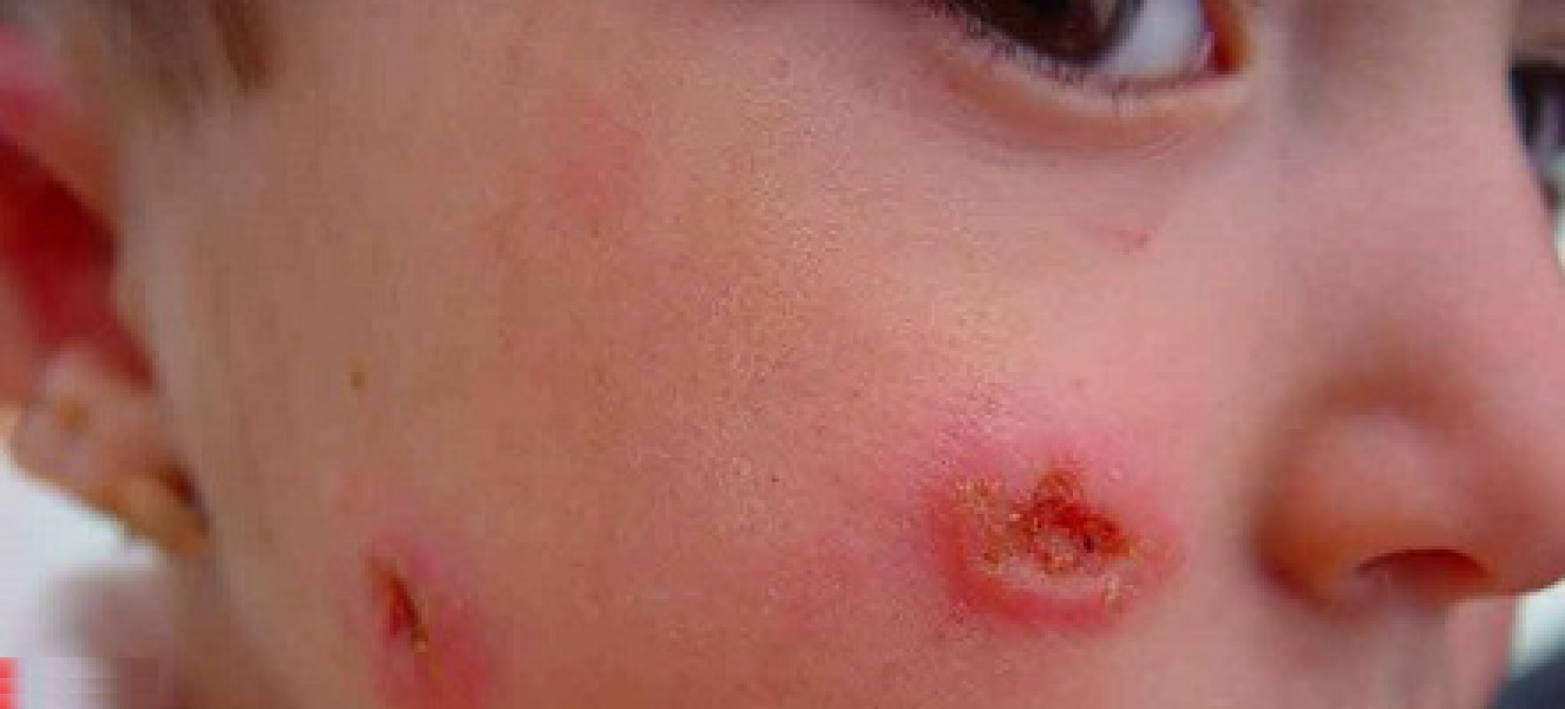
Cutaneous leishmaniasis (Cl), example 2

Dear Editor, 

Leishmaniasis is a neglected tropical disease caused by a parasite transmitted by infected female sandflies. There are 3 main forms of the disease [1]:

Cutaneous leishmaniasis (Cl) is the most common form and causes skin lesions, mainly ulcers, on exposed parts of the body (see examples in Figure 1 (Fig. 1) and Figure 2 (Fig. 2)). It is estimated that 600,000 to 1 million new cases occur worldwide annually. 

Mucocutaneous leishmaniasis leads to partial or total destruction of mucous membranes of the nose, mouth and throat. Over 90% of mucocutaneous leishmaniasis cases occur in Bolivia, Brazil, Ethiopia and Peru.

Visceral leishmaniasis, also known as kala-azar, is fatal if left untreated in over 95% of cases. Most cases occur in Brazil, east Africa and India. An estimated 50,000 to 90,000 new cases occur worldwide annually. Visceral leishmaniasis is highly endemic in Iraq, Somalia, Sudan and Yemen.

Several Leishmania spp. can cause Cl, but most infections probably remain symptomless. The first sign of an infection is typically a small erythema, which develops into a papule, then a nodule that progressively ulcerates over a period of 2 weeks to 6 months. Lymphatic spread and lymph-gland involvement, which may precede lesion development, are common and there is a variable tendency for lesions to self-heal [2]. Resolution of Cl, but also the other forms of leishmaniasis with skin involvement, results in a lifelong cutaneous scar, which can cause permanent disfiguring skin lesions that may last a lifetime, leading to physical limitations, social isolation, and feelings of embarrassment [3]. In addition to these physical effects, leishmaniasis can have profound psycho-social effects on individuals, including feelings of shame, social exclusion, and stigmatization. These effects can further exacerbate the negative impact of the disease on quality of life, making it important for healthcare providers to take a comprehensive approach to managing and treating leishmaniasis [4]. 

Healthy skin is essential for physical well-being and can have a significant impact on an individual's self-reliance and sexual attractiveness. Cl can make life very difficult for affected individuals, as the visible skin lesions can create emotional distress and make routine relationships with close relatives and friends difficult, particularly when the lesions are visible in exposed parts of the body.

In addition to the psycho-social effects of visible lesions, the permanent scars left by the disease can negatively impact an individual's quality of life, creating social issues and psychological symptoms. As such, it is important for healthcare providers to take a holistic approach to managing and treating leishmaniasis, addressing both the physical and psycho-social aspects of the disease to improve outcomes for affected individuals [5].

Quality of life is a subjective perception that is influenced by an individual's values, culture, goals, standards, expectations, and concerns. This concept is particularly relevant in chronic diseases like leishmaniasis, as it can help capture the disease's social and psychosomatic impact and the effectiveness of therapeutic interventions. By taking a comprehensive approach to the management and treatment of leishmaniasis, healthcare providers can improve the quality of life for affected individuals [6]. 

Assessing patients’ quality of life with Cl can improve our understanding of their needs and the psycho-social consequences of the disease. This can help develop effective therapeutic solutions, improving outcomes for affected individuals. By taking a patient-centred approach, healthcare providers can optimize outcomes and improve the overall quality of life for those with leishmaniasis. This may involve tailored treatment plans and ongoing support and education for patients to manage the disease and its effects on their daily lives [7]. 

## Conclusions

Cl can have significant physical and psychological impacts on patients’ quality of life. To create a better quality of life for affected individuals, healthcare professionals should consider all aspects of the disease in conjunction with therapeutic interventions. 

In addition, evaluating the extent of disease involvement and characteristics such as the number, size, and duration of scars, along with potential side effects of treatment and residual lesions, is crucial for planning a successful patient care management program. By taking a comprehensive approach that addresses the disease’s physical and psycho-social aspects, healthcare providers can improve outcomes and quality of life for those with Cl.

## Addendum of the editor

For countries where Cl is not endemic, it is important to know where leishmaniasis is endemic in order to be aware of the disease in travellers returning with symptoms. About 95% of Cl cases occur in the Americas, the Mediterranean basin, the Middle East and central Asia. Cl is highly endemic in Algeria, whereas for West Africa, the epidemiological information is scarce. In East Africa, all forms are endemic. In the following countries leishmaniasis is endemic [8]:

Afghanistan, Albania, Algeria, Argentinia, Azerbajan, Belize, Bhutan, Bolivia, Bosnia and Herzegovina, Brazil, Bulgaria, Burkina Faso, Cameroon, Central African Republic, Chad, China, Colombia, Costa Rica, Croatia, Cyprus, Côte d’Ivoir, Democratic Republic Congo, Djbouti, Dominican Republic, Ecuador, Egypt, El Salvador, Eritrea, Ethiopia, France, French Guiana, Gambia, Georgia, Ghana, Greece,Guatemala, Guinea, Guinea-Bissau, Guyana, Honduras, India, Iran, Iraq, Israel, Italy, Jordan, Kazakhstan, Kenya, Kuwait, Kyrgyzstan, Lebanon, Libya, Malawi, Mali, Malta, Mauritania, Mexico, Monaco, Montenegro, Morocco, Namibia, Nepal, Nicaragua, Niger, Nigeria, Oman, Pakistan, Panama, Paraguay, Peru, Portugal, Republic of North Macedonia, Saudi Arabia, Senegal, Slovenia, Spain, Sri Lanka, Siudan, Suriname, Syrian Arab Republic, Taiwan, Tajikistan, Thailand,Tunisia, Turkmenistan, Turkey, Ukraine, USA, Uzbekistan, Venezuela, Palestine, and Yemen. 

In northern and central Europe, no authochthonus cases have been reported. That is, in Germany, leishmaniasis occurs almost as an imported disease. In 2000, a reference centre for the diagnosis and therapy of leishmaniasis was opened at the Institute for Tropical Medicine, Berlin, Germany. During the first two years, 58 cases of leishmaniasis were imported [9]. Up to 2007, 130 imported leishmaniasis cases had been diagnosed, of which 91 were cutaneous, 5 were mucosal and 34 were visceral forms. 38% of cutaneous and 97% of visceral infections in German travellers originated from Southern Europe, the distribution area of leishmania infantum. 42% of the patients contracted cutaneous leishmaniasis in Latin America [10]. As a consequenc of climate change, however, an increase in autochthonous cases in Germany is to be expected, because in 1998 the first sandflies were detected in Germany in the Upper Rhine Valley [11]. In the same year, a visceral leishmaniasis in a 15-month-old German child was diagnosed, who had no history of travel to areas known to be endemic for leishmaniasis [12]. Others described cases of canine and equine leishmaniasis exist near Landsberg/Lech and Cologne [13], [14].

## Notes

### Competing interests

The authors declare that they have no competing interests.

### Funding 

Not applicable

### Author’s ORCID

The ORCID ID of Azami M is: 0000-0003-2794-1508
